# Clinical efficacy of acupuncture combined with Western medicine in the treatment of mild to moderate Alzheimer disease: A protocol of a randomized controlled trial

**DOI:** 10.1097/MD.0000000000030705

**Published:** 2022-09-30

**Authors:** Kangmei Zhou, Jierong He, Lin Quan, Rong Guo

**Affiliations:** a Deyang Vocational College of Technology and Trade, Deyang, China.

**Keywords:** acupuncture, Alzheimer disease, protocol, randomized controlled trial

## Abstract

Alzheimer disease (AD) is a common cause of dementia, and there are still a lack of treatment options to reverse or prevent disease progression. Existing evidence shows that acupuncture has advantages in the treatment of AD, but whether the efficacy of acupuncture belongs to the placebo effect remains controversial, and there is no strict placebo-controlled clinical study to evaluate the efficacy and safety of acupuncture combined with Western medicine in the treatment of AD.

**Methods:**

This is a prospective randomized, a single-blind, sham-acupuncture controlled trial to study the clinical efficacy of acupuncture combined with Western medicine in the treatment of mild to moderate AD. Participants will be randomly divided into treatment and control groups. The treatment group using acupuncture combined with donepezil hydrochloride orally, and the control group using sham acupuncture combined with donepezil hydrochloride orally, followed up for 24 weeks after 24 weeks of continuous treatment. Outcome measures included: AD assessment scale-cognitive subscale, mini-mental state examination, activities of daily living, neuropsychiatric inventory questionnaire, serum superoxide dismutase, and homocysteine levels. Finally, SPASS 21.0 software was used for statistical analysis of the data.

**Discussion:**

This study will evaluate the efficacy of acupuncture combined with Western medicine in improving cognitive function and activities of daily living in AD patients. The results of this study will verify whether the efficacy of acupuncture in the treatment of AD belongs to the placebo effect, which will also provide a reference for the clinical use of acupuncture combined with Western medicine in the treatment of AD.

**Trial registration:**

The TCTR identification number is TCTR20220817004.

## 1. Introduction

Alzheimer disease (AD) is a common cause of dementia, and the main clinical manifestations are aphasia, agnosia, and gradual loss of independent living ability.^[1]^ With the acceleration of the aging process of the population, the incidence rate of AD continues to rise. And the mortality rates of major diseases such as AIDS, stroke, and heart disease in the elderly in the United States have decreased over the past 15 years, with only AD increasing by 71%.^[2]^ The onset of the disease is insidious and progressively aggravated, generally with obvious symptoms after 2 to 3 years of onset, gradual memory, cognitive dysfunction, and behavioral abnormalities, and death due to complications such as pulmonary and urinary tract infections and pressure sores 10 to 20 years after onset.^[3]^ The pathogenesis of this disease is complex, and there is no effective drug that can reverse the disease process.^[4]^ It is important to take timely and effective therapeutic drugs and measures to delay the development of the disease.

Although researchers have made great efforts for the study of AD, there is no specific therapeutic drug at present. Cholinesterase inhibitors and memantine are drugs approved by the Food and Drug Administration and European Agency for the Evaluation of Medical Products for the treatment of AD, and although they can improve cognitive function and delay the disease in patients,^[5]^ they are not effective alone and have adverse effects on long-term use.^[6]^

Acupuncture and moxibustion are traditional treatments in China and are widely used in clinical practice. In the treatment of AD, it has the characteristics of multiple levels, multiple pathways, and multiple targets, and the advantages of reliable efficacy and few side effects.^[7,8]^ Recent systematic reviews have shown^[9,10]^ that acupuncture combined with Western medicine is superior to Western medicine alone in improving cognitive function and activities of daily living in AD patients. At the same time, these systematic reviews also point out that there are serious methodological shortcomings in current randomized controlled trials on acupuncture treatment of AD, such as randomization problems, lack of sample size estimation, and no blind method.^[9–11]^ These deficiencies also lead to a low strength of evidence for the research results, limiting the application of acupuncture in AD. Limited by acupuncture treatment modalities, strict double-blind cannot be achieved in clinical practice,^[12]^ and whether its clinical efficacy belongs to the placebo effect is controversial.^[13,14]^ At present, domestic and foreign scholars are continuously exploring the optimization of placebo acupuncture in order to explore the specific effect of acupuncture.^[15]^ This study will study the clinical efficacy of acupuncture combined with Western medicine in the treatment of mild to moderate AD by randomized, single-blind, placebo acupuncture parallel control method.

## 2. Materials and Methods

### 2.1. Study design

**Figure 1.**
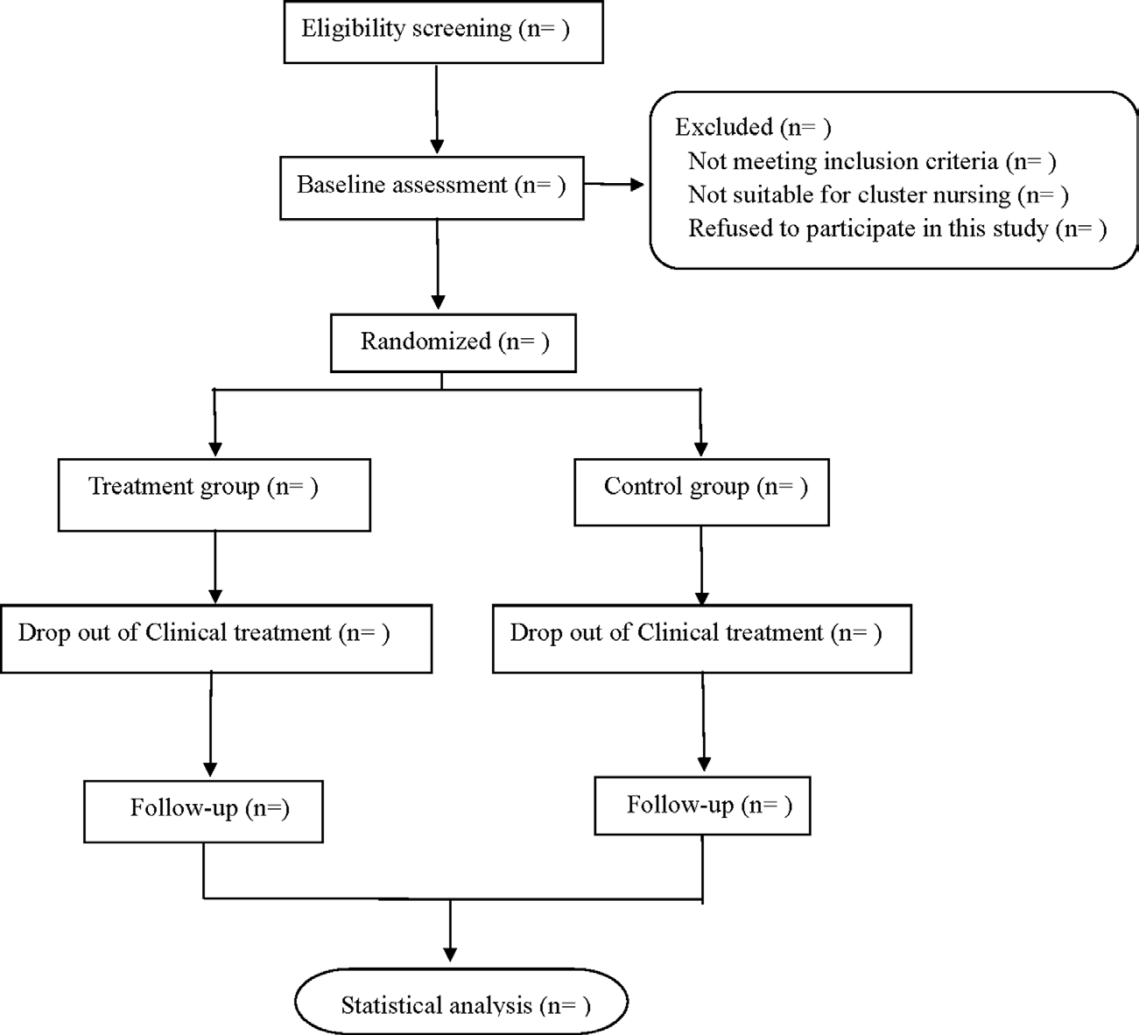
Flow diagram.

This is a prospective, randomized, single-blind, sham acupuncture controlled trial to study the clinical efficacy of acupuncture combined with Western medicine in the treatment of mild to moderate AD. Participants will be randomly divided into treatment and control groups. The treatment group will receive oral donepezil hydrochloride combined with acupuncture treatment, and the control group will receive oral donepezil hydrochloride combined with sham acupuncture treatment. After 24 weeks of continuous treatment, the participants will be followed up for 24 weeks. Flow diagram is shown in Figure 1, and study schedule is shown in Table 1. This experiment will follow the standard for reporting intervention in acupuncture controlled trials^[16]^ and the standard for reporting comprehensive trials.^[17]^

**Table 1:** Study schedule.

Project	Stage				
	Screening period	Treatment period		Follow-up	
	Baseline	12-wk	24-wk	12-wk	24-wk
Record fill	√				
Fulfill inclusion criteria and exclusion criteria	√				
Sign informed consent	√				
Random allocation	√				
Treatment	√	√	√		
Effectiveness observation					
ADAS-Cog	√	√	√	√	√
MMSE	√	√	√	√	√
ADL	√	√	√	√	√
NPI	√	√	√	√	√
SOD and Hcy	√	√	√	√	√
Safety evaluation					
Blood test and urinalysis	√		√		√
Liver and kidney function	√		√		√
Record of adverse event		√	√	√	√

ADAS-Cog = Alzheimer disease assessment scale-cognitive subscale, ADL = activities of daily living, Hcy = homocysteine, MMSE = mini-mental state examination, NPI = neuropsychiatric inventory questionnaire, SOD = serum superoxide dismutase.

### 2.2. Ethics and registration

The study protocol will be conducted in accordance with the Declaration of Helsinki and Ethical Guidelines for Clinical Research. This study has been reviewed by the Clinical Research Ethics Committee of our hospital and registered on the Thai Clinical Trials Registry (registration number: TCTR20220817004). Before randomization, all patients were asked to sign an informed consent form and were given the option whether to continue the trial at any time.

### 2.3. Patients

#### 2.3.1. Diagnosis basis.

The diagnostic standard for AD refers to the diagnostic standard for AD established by the National Institute on Aging-Alzheimer’s Association workgroups on diagnostic guidelines for AD in the United States.^[18,19]^ TCM diagnostic standard refers to Chinese disease diagnostic efficacy standards.^[20]^

#### 2.3.2. Inclusion criteria.

1. Meet the diagnostic standard of Alzheimer disease.
2. Aged ≥50 years old and ≤85 years old.
3. Patients with mild to moderate Alzheimer disease (dementia degree judged as mini-mental state examination score 10–23 points)^[21]^.
4. Patients recognize the study protocol and sign the informed consent form.

#### 2.3.3. Exclusion criteria.

1. There are other diseases that may lead to dementia besides Alzheimer disease.
2. Acute cardiovascular and cerebrovascular events (such as unstable angina pectoris, myocardial infarction, stroke, etc.) occurred within 3 months before screening.
3. There was skin ulceration at acupuncture points.
4. Refusal or fear of acupuncture.
5. Those who have participated in or are participating in other clinical trials within the past 1 month.

#### 2.3.4. Standard for failing off and suspension.

1. The patient withdrew from the study for any reason.
2. The principal investigator considered that the subject was not suitable for continuing the study.
3. The subjects had poor compliance and changed the drug or added unspecified therapeutic drugs by themselves, which had an impact on the study results.
4. Serious safety events occurred during the study and were assessed to be inappropriate for continuing the study.
5. The patient was accidentally included after misdiagnosis or did not meet the inclusion and exclusion standard.

For all dropouts and patients who discontinue the trial, we will complete the last data test as far as possible. The dropout reasons and handling methods for all dropouts will be recorded on the case report form (CRF).

### 2.4. Sample size

The sample size calculation in this study was based on the improvement of the score of the AD assessment scale-cognitive subscale (ADAS-Cog)^[22]^ after the end of treatment (the difference of the mean standard deviation of the ADAS-Cog score before and after treatment), referring to the results of the pretest, 6.65 ± 3.23 in the treatment group and 4.51 ± 2.11 in the control group, using the difference test, setting *α* = 0.05, *β* = 0.10. PASS15.0 (NCSS Company, Kaysville, UT) software calculated that 36 patients were needed in each group. The estimated withdrawal rate was 10%, and the total sample size required was 80 patients, 40 patients in each group.

### 2.5. Randomization and blinding

Patients meeting the inclusion and exclusion standard will be randomized in a 1:1 ratio by a central web-based randomization tool to either the treatment group (acupuncture combined with oral donepezil hydrochloride) or the control group (sham acupuncture combined with oral donepezil hydrochloride). Random sequences were generated by an independent statistician using SAS 9.3 software (SAS Institute, Cary, NC). Research assistants entered patient information on a tablet computer and were assigned a random number to complete the random assignment based on the random number. Limited by the way of acupuncture, acupuncture operators were aware of the grouping results, and patients, other investigators, and data statisticians were not aware of the grouping results.

### 2.6. Intervention measures

Based on the recommendations of the EFNS guidelines for the diagnosis and management of Alzheimer disease,^[23]^ patients in two groups will receive oral donepezil hydrochloride 5 mg at bedtime (Weicai Pharmaceutical Co., Ltd., Shanghai, China, GYZZ H20050978, 5 mg/tablet) once a day for 24 weeks.

#### 2.6.1. Acupuncture treatment.

This study will follow the international acupuncture clinical trial standard STRICTA to ensure the quality control of acupuncture treatment.^[24]^ Acupuncture points include: Baihui (GV20), Sishencong (EX-HN1), Dazhui (DU14), Shenmen (HT7), Shenshu (BL23), and Taixi (KI3).

To ensure the implementation of blinding, patients in both groups will wear eye masks during acupuncture, take the supine position, and the acupoints will be disinfected using 75% alcohol. An acupuncturist with a TCM license and at least 3 years of clinical experience will complete acupuncture treatment using a disposable stainless steel acupuncture needle (0.25 mm × 40 mm; Suzhou Hualun Medical Appliance Co., Ltd, Suzhou, China). The treatment group required acupuncture “Needling Sensation” (Deqi), and 0.5 cm non-acupoint needle insertion in the control group at a depth of 1 to 2 nn without requiring “Needling Sensation” (Deqi).^[25]^ Every other day for 30 minutes for 24 weeks.

### 2.7. Outcomes

#### 2.7.1. Primary outcomes.

ADAS-Cog^[22]^ evaluates cognitive function: including memory, ability to use, orientation, language expression ability, attention, reasoning ability and other items, and the higher the score, the more severe the neurological impairment.

#### 2.7.2. Secondary outcomes.

1. Mini-mental state examination^[26]^ evaluates mental status, including five items: orientation; memory; attention and calculation, memory; and language ability.
2. Activities of daily living^[22]^ assesses patients’ living ability: including 6 aspects related to physical self-care and 8 aspects related to the ability to use tools.
3. Neuropsychiatric inventory questionnaire^[27]^ evaluates common mental and behavioral symptoms of dementia: including 12 items such as delusions, hallucinations, agitation, and unpleasant mood.
4. Serum superoxide dismutase and homocysteine levels: Fasting venous blood was collected from the two groups in the morning before and after treatment. Serum superoxide dismutase levels were measured by enzyme-linked immunosorbent assay, and homocysteine levels were measured by circulating enzymatic method.

Two research assistants will collect data based on the above outcome measures at baseline, 12 weeks after treatment, and at the end of treatment. All patients will be followed for 24 weeks and efficacy evaluations will be completed in the clinic at Weeks 12 and 24 of follow-up.

### 2.8. Safety evaluation

Patients will be asked to report any abnormal reactions that occur during the study to the investigator. The details of all adverse events will be recorded in the CRF, including the occurrence time, the degree and duration, suspected cause, effective measures, and results. Blood routine, urine routine, and liver and kidney function were measured at baseline and at the end of treatment. Safety measures were assessed in all patients at the end of the study to assess the safety of the study protocol.

### 2.9. Data management and quality control

Any amendment or change to the protocol will be re-approved through the official procedure of our hospital ethics committee. An independent clinical research associate will periodically review the progress of the study. Trained study personnel will collect study data and record it on the CRF. To ensure the reliability of the data, personal information about potential and registered participants will be collected, shared, and kept in an independent repository to protect confidentiality before, during, and after the trial. Access to the database will be restricted to study personnel on this study team. Participants’ information will not be made publicly available and shared without written permission from participants.

### 2.10. Statistical analysis

The study results will be independently analyzed by Full Analysis Set and Per-Protocol Set, and the safety evaluation will be based on Safety Set. Statistical evaluation of Full Analysis Set will follow intent-to-treat. Missing values will be measured using the last observation carried forward method. Data from this study will be statistically analyzed using SPSS 21.0 software (IBM Company, New York, NY). Count and grade data were expressed as percentage (%), the measurement data of *χ*^2^ test or non-parametric test were expressed as mean ± standard deviation (*x* ± S) or interquartile range M (P25, P75), normal distribution was analyzed by *t* test, skewed distribution was analyzed by non-parametric test, and One-way analysis of variance multiple comparisons were used between multi-point repeated data groups, and differences were considered statistically significant when *P < *.05.

## 3. Discussion

AD is a chronic progressive neurodegenerative disease characterized by cognitive impairment such as memory impairment and personality changes.^[7]^ Modern studies have found that abnormal cholinergic neurons in the basal forebrain leading to decreased acetylcholine function in the hippocampus are the main pathological factors causing AD, but also cause behavioral and cognitive changes in patients, causing memory and orientation decline.^[28]^ Donepezil hydrochloride is a second-generation specific reversible central acetylcholinesterase inhibitor, which can inhibit acetylcholoyl hydrolysis, improve cognitive function, and alleviate hippocampal atrophy in patients.^[29]^ Clinical studies have found limited efficacy of donepezil hydrochloride alone in the treatment of AD and advocate the use of combined therapeutic regimens.^[30]^

As an important complementary and alternative therapy, acupuncture has a reliable effect in improving cognition and promoting brain function recovery.^[31,32]^ Modern studies have shown that acupuncture can enhance functional connectivity between hippocampal regions and activate specific cognitive related areas of the brain.^[33]^ Acupuncture can also reduce oxidative stress response and enhance oxygen-free radical metabolism capacity.^[34]^ Although some clinical studies have shown that acupuncture is reliable in the treatment of AD. These studies are generally of low quality and lack long-term effective follow-up. This study will avoid the effect of placebo effect on the study results by means of sham acupuncture control, and we also increased the follow-up time to observe the long-term effect of acupuncture combined with western medicine on mild to moderate AD.

This study also has some shortcomings: due to the limitation of intervention methods, this study will not achieve strict double-blind, which may have a certain impact on the results; this study will be conducted in a hospital in China, so there may be regionalization of the subject population, which biases the results.

## Author contributions

**Data curation:** Kangmei Zhou and Jierong He.

**Formal analysis:** Kangmei Zhou and Jierong He.

**Funding support:** Rong Guo.

**Software:** Lin Quan.

**Supervision:** Rong Guo.

**Writing – original draft:** Kangmei Zhou and Jierong He.

**Writing – review & editing:** Rong Guo.
